# Increases in humidity will intensify lethal hyperthermia risk for birds occupying humid lowlands

**DOI:** 10.1093/conphys/coaf036

**Published:** 2025-06-03

**Authors:** Bianca Coulson, Marc T Freeman, Shannon R Conradie, Andrew E McKechnie

**Affiliations:** South African Research Chair in Conservation Physiology, South African National Biodiversity Institute, 2 Cussonia Ave, Brummeria, Pretoria 0184, South Africa; Department of Zoology and Entomology, University of Pretoria, Lynnwood Rd., Pretoria 0002, South Africa; South African Research Chair in Conservation Physiology, South African National Biodiversity Institute, 2 Cussonia Ave, Brummeria, Pretoria 0184, South Africa; Department of Zoology and Entomology, University of Pretoria, Lynnwood Rd., Pretoria 0002, South Africa; School of Animal, Plant, and Environmental Sciences, University of the Witwatersrand, 1 Jan Smuts Ave, Braamfontein, Johannesburg 2001, South Africa; South African Research Chair in Conservation Physiology, South African National Biodiversity Institute, 2 Cussonia Ave, Brummeria, Pretoria 0184, South Africa; Department of Zoology and Entomology, University of Pretoria, Lynnwood Rd., Pretoria 0002, South Africa

**Keywords:** Body temperature, evaporative cooling, forest, heat tolerance limit, hornbill, humidity, thermoregulation

## Abstract

Increasingly frequent and intense heatwaves are expected to elevate the risks of heat-related mortality among birds. Most studies have focused on arid-zone avifaunas and the extent to which risks will increase in other habitats, particularly humid lowlands, remains unclear. We tested the prediction that increasing air temperature and corresponding increases in humidity, and hence wet-bulb temperature (*T*_W_; lowest temperature achievable via adiabatic evaporation), will increase exposure to conditions associated with lethal hyperthermia. We empirically determined maximum *T*_W_ (*T*_W-max_) for an Afrotropical forest frugivore, the trumpeter hornbill (*Bycanistes bucinator*) as *T*_W-max_ = 31.7 ± 1.0°C. We then modelled current and future exposure to conditions associated with *T*_W_ > *T*_W-max_ across this species’ range. Under a business-as-usual emissions scenario and assuming no vegetation buffering of air temperature (*T*_air_), trumpeter hornbills will experience *T*_W_ > *T*_W-max_ for at least 1 day year^−1^ over 46% of their current range, compared to 30% at present. However, the frequency of exposure will increase substantially and reach ~100 days year^−1^ in parts of the southern Democratic Republic of Congo. When we incorporated the thermal buffering effect of vegetation, end-century exposure to *T*_W_ > *T*_W-max_ decreased by 0.3–66.7%, emphasizing the role of cool microsites provided by vegetation. Our analyses reveal the exposure of birds inhabiting humid environments at low latitudes to conditions associated with a risk of lethal hyperthermia under resting conditions will increase substantially in coming decades, putting a large fraction of global avian biodiversity at risk of population declines and local extinctions.

## Graphical Abstract

**Graphical Abstract:**
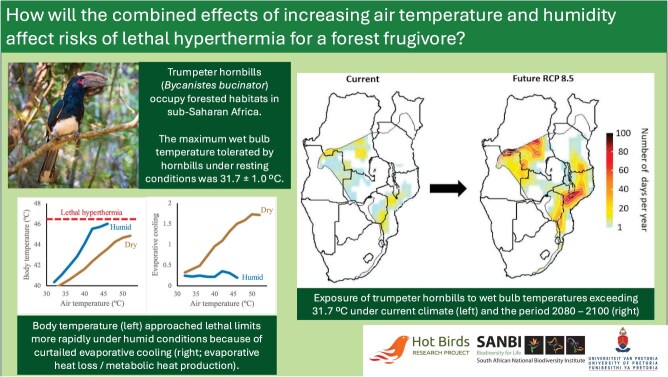

## Introduction

Approximately 72% of bird species occupy forest habitats at tropical and subtropical latitudes (Pillay *et al.,* 2022), making an understanding of how climate change will affect avian biodiversity in these regions essential for global conservation efforts. The climates associated with tropical and subtropical forests, particularly those in lowlands including many coastal regions, are characterized by a combination of maximum air temperatures (*T*_air_) that approach avian normothermic body temperature (*T*_b_) and high atmospheric humidity. Elevated humidity impedes evaporative heat dissipation, reducing avian thermoregulatory performance during hot weather (Lasiewski *et al.,* 1966; Powers, 1992; Gerson *et al.,* 2014; Freeman *et al.,* 2024) and increasing the risk of heat-related mortality for taxa including birds (Gerson *et al.,* 2014; McKechnie *et al.,* 2021b) and pteropodid bats (Welbergen *et al.,* 2008; Ratnayake *et al.,* 2019). Although evidence is accumulating that high humidity has shaped the evolution of the thermal physiology of birds occupying humid lowlands (Weathers, 1997; Freeman *et al.,* 2022; Freeman *et al.,* 2024), the increases in *T*_air_ and humidity predicted for many regions, including large parts of the tropics and subtropics during the 21st century (Willett *et al.,* 2007; Douville *et al.,* 2022; IPCC, 2023), may expose forest species to potentially lethal conditions more frequently than has been the case in the past.

Assessments of how lethal effects of acute heat exposure during heat waves will increase among birds in coming decades have largely focused on species inhabiting arid regions, with models based on projected increases in *T*_air_ alone (Albright *et al.,* 2017; Conradie *et al.,* 2019; Conradie *et al.,* 2020). However, the interacting effects of humidity and *T*_air_ on avian thermoregulation in humid environments necessitate a different approach for modelling hyperthermia risk under anticipated future climates. Over the last decade, analyses of upper limits to human outdoor survival have increasingly focused on wet-bulb temperature (*T*_W_), the lowest temperature achievable by adiabatic evaporation of water into air and measured by enclosing a thermometer’s bulb in a wet wick (Campbell and Norman, 1998). An adaptability limit for young, healthy adults at *T*_W_ = 35°C, above which hyperthermia becomes unavoidable, was proposed by Sherwood and Huber (2010), although recent arguments have been made for a lower threshold of *T*_W_ = 31°C (Vecellio *et al.,* 2022).

Whereas risks of lethal hyperthermia to humans under current and future climates are now routinely modelled using threshold values of *T*_W_ (e.g. Pal and Eltahir, 2016; Coffel *et al.,* 2017; Raymond *et al.,* 2020), the same is not true for non-human species. We propose that ecologically relevant maximum *T*_W_ (*T*_W-max_) for resting birds and other animals during acute heat exposure should meet the conditions of 1) severely diminished evaporative cooling capacity, evident as an inability to maintain *T*_b_ below environmental temperature and 2) severe hyperthermia, evident as compromised locomotor function such as balance or righting response. Although avian thermoregulation at environmental temperatures exceeding normothermic *T*_b_ has been a topic of long-standing interest (Dawson, 1954; Tieleman *et al.,* 2002; McKechnie *et al.,* 2021a; Freeman *et al.,* 2024), we are not aware of published data suitable for estimating avian *T*_W-max_ values under resting conditions.

An important consideration for predicting climate change impacts on forest birds is that exposure to high *T*_W_ may be buffered by cool, shaded microclimates in forest interiors. The *T*_air_ within tropical forests is typically lower than outside (De Frenne *et al.,* 2021), with understory *T*_air_ at midday often >5°C cooler (e.g. Fetcher *et al.,* 1985; Ghuman and Lal, 1987; Chow *et al.,* 2016). Moreover, ground-level *T*_air_ is typically 0.5–2.5°C cooler than canopy temperatures (Madigosky, 2004), although the cooling effect of evapotranspiration is reduced at high relative humidity (Davis *et al.,* 2019). Absolute humidity is generally similar between forest interiors and the areas surrounding forests, but can be lower within forests when large *T*_air_ gradients result in near-saturated interior conditions (e.g. Fetcher *et al.,* 1985; Ghuman and Lal, 1987).

Here, we quantified risks of lethal hyperthermia under recent and future climates in hot, humid forest habitats using a ~650-g Old World forest frugivore, the trumpeter hornbill (*Bycanistes bucinator,* Temminck 1824) as a model species. Hornbills (Bucerotiformes: Bucerotidae) are prominent avian frugivores in the forests of Africa and Asia, with trumpeter hornbills occurring in forested habitats in southern and eastern Africa (Kemp and Woodcock, 1995). The potential loss of large avian frugivores with advancing climate change is of particular concern on account of the seed dispersal ecosystem services they provide and their role in forest persistence and gene flow in fragmented habitats (Moran *et al.,* 2004; Mueller *et al.,* 2014). We first evaluated the effects of humidity on thermoregulation in *B. bucinator* by comparing relationships between *T*_air_, metabolic heat production, evaporative heat loss and *T*_b_ over a range of experimental humidities. We used these data to empirically determine *T*_W-max_ for the species and then modelled exposure to conditions of *T*_W_ > *T*_W-max_ under recent and anticipated future climates to test two predictions. First, we predicted that increasing *T*_air_ associated with anthropogenic global heating and corresponding increases in humidity will increase exposure to conditions of *T*_W_ > *T*_W-max_ and thus the risk of lethal hyperthermia. Second, we predicted that cool, shaded microclimates within forest interiors reduce forest birds’ exposure to conditions of *T*_W_ > *T*_W-max_.

## Materials and Methods

### Study site and species

We captured 28 trumpeter hornbills (hereafter, hornbills) using mist nets (Avinet Nylon 100-mm mesh, Denier/ply 210/4, 5 shelves, 3 m height) in the lowland coastal forests of KwaZulu-Natal province, South Africa, in and around the towns of St Lucia, Richard’s Bay and Mtunzini (S 28.373°, E 32.414°–S 28.960°, E 31.750°) between October and December 2022. Our study site is within the Indian Ocean coastal belt biome (Mucina and Rutherford, 2006) with mean austral spring/summer (September–March) maximum *T*_air_ = 28.0 ± 1.9°C, minimum *T*_air_ = 19.4 ± 1.2°C and mean annual precipitation of ∼1190 mm in the study area. Mean spring/summer humidity is 18.44 + 0.65 g H_2_O m^−3^, equivalent to a dewpoint of 21.9°C or a relative humidity of 46.7% at *T*_air_ = 35°C (Fick and Hijmans, 2017).

Following capture, birds were transported in clean, large cotton pillowcases (for ≤1 h) to temporary outdoor aviaries (2 × 2 × 2 m) where they were housed in family groups whenever >1 individual was held at a time. Following respirometry measurements detailed below, individuals remained in captivity for 3–4 days to assess condition and for thermal imaging for a separate study. Birds were provided with *ab libitum* water and soft fruits (mainly papaya) throughout the day. Birds showed no obvious adverse effects, and an individual re-trapped several weeks after measurements was in good health. We obtained data for 16 males and 12 females [mean ± SD body mass (*M*_b_): 748 ± 130 g and 580 ± 111 g, respectively; Table S1]. Prior to experiments, a temperature-sensitive passive integrated transponder (PIT) tag (Biotherm 13, Biomark, Boise, ID, USA) was injected subcutaneously into the abdominal cavity of each hornbill for body temperature (*T*_b_) measurements following Czenze *et al.* (2020) and Freeman *et al.* (2022, 2024). Two reader–transceiver systems (HPR+, Biomark, Boise ID, USA) placed against the chamber were used to read *T*_b_ values from the PIT tags.

#### Effects of humidity on thermoregulation and estimation of maximum wet-bulb temperature

We quantified hornbills’ physiological responses across the approximate range of *T*_W_ they experienced naturally during the austral summers (October–March) of 2002–22, calculated from South African Weather Service data for Richard’s Bay (S 28.737°, E 32.093°). We calculated *T*_W_ using the equation proposed by Stull (2011):

*T*_W_ = *T*_air_ atan[0.151977(RH + 8.313659)^1/2^] + atan(*T*_air_ + RH) – atan(RH − 1.676331) + 0.00391838RH^3/2^ atan(0.023101RH) − 4.686035.

where RH is relative humidity (%). Several methods of calculating *T*_W_ are available and the values calculated using Stull’s equation can differ by ~1°C from those estimated using the iterative procedure described by Davies-Jones (2008) (Vecellio *et al.,* 2023). We opted to use Stull’s (2011) equation as it is computationally simpler and produces the same *T*_W_ as many online *T*_W_ calculators available to conservationists, protected area managers and the general public. Moreover, the method used for calculating *T*_W_ does not affect our quantitative or qualitative conclusions, as it was used for calculating both environmental *T*_W_ and the hornbills’ T_W-max_.

We measured *T*_b_, evaporative water loss (EWL) and metabolic heat production (MHP) of hornbills resting in metabolic chambers at *T*_air_ between 28 and 52°C at one or more of three experimental humidity levels: ~6 g H_2_O m^−3^, ~13 g H_2_O m^−3^ and ~25 g H_2_O m^−3^, equivalent to dewpoints of ~5°C, 16°C and 27°C, respectively. Our experimental setup and measurement techniques were the same as those of Freeman *et al.* (2024), with modifications listed below. Hornbills were placed individually in a sealed 30-L chamber (62 cm long × 32 cm wide, with a sloping lid such that the height varied from 40 to 32 cm) constructed from transparent polycarbonate and fitted with a mesh platform ~10 cm above a 1- to 2-cm deep mineral oil layer to prevent evaporation from excreta from affecting EWL measurements.

Humidity in the respirometry chamber was regulated following Freeman *et al*. (2024 – also see Supplementary Methods for further details) at either ~6, ~13 or ~25 g H_2_O m^−3^, hereafter referred to as the low, intermediate and high humidity treatments, respectively. This experimental design meant that the evaporative gradient (i.e. difference in absolute humidity between saturated and experimental conditions) varied between the various combinations of *T*_air_ and humidity we used (Campbell and Norman, 1998), as is the case for free-ranging hornbills. Under natural conditions, hornbills in the study area sometimes experience humidity higher than our maximum experimental value, but the practicalities of manipulating in-chamber humidity while ensuring no condensation in the respirometry system made 25 g H_2_O m^−3^ the highest experimental humidity feasible in our study.

Each set of measurements lasted 2–6 h and began with a bird placed in the chamber at *T*_air_ = 28°C and allowed 1 h to habituate to experimental conditions. To avoid condensation in the chamber and tubing and ensure the target humidity was reached, measurements would begin at *T*_air_ = 28°C for the low treatment, *T*_air_ = 30°C for the intermediate treatment and *T*_air_ = 32°C for the high treatment. For the low-humidity treatment, *T*_air_ was increased incrementally by 4°C to *T*_air_ = 40°C, after which *T*_air_ was increased in 2°C increments until the birds reached thermal endpoints. For the intermediate- and high-humidity treatments, *T*_air_ was incrementally increased by 2°C from the starting *T*_air_ until thermal endpoints were reached. Thermal endpoints were identified by rapid, unregulated increases in *T*_b_, loss of coordination or rapid declines in EWL or resting metabolic rate (RMR) (Whitfield *et al.,* 2015). For all protocols, birds were exposed to each *T*_air_ setpoint for 25–30 min. Transitions between *T*_air_ setpoints for all protocols took 20–25 min, and at each setpoint, birds were exposed to stable *T*_air_ and humidity for a minimum of 15–20 min until traces of CO_2_ and H_2_O were stable for at least 5 min.

#### Ethics

This work was approved by the Animal Ethics Committee of the University of Pretoria (NAS063/2022) and the Research and Scientific Ethics Committee of the South African National Biodiversity Institute (SANBI NZG/RES P2022/16). Birds were captured under permit OP 3026/2022 from KwaZulu-Natal province’s Ezemvelo KZN Wildlife office.

#### Analyses of effects of humidity on thermoregulation

All statistical analyses were conducted in the R environment (v4.2.2) using RStudio. Using the ‘segmented.lme’ function from the *segmented* package (Muggeo, 2016) inflection *T*_air_ values above which *T*_b_, RMR, EWL and evaporative heat loss/metabolic heat production (EHL/MHP) increased rapidly for each humidity treatment were identified, with individual identity included as a random effect. Model selection was conducted using the ‘model_sel’ function in the *MuMIn* package (Bartoń, 2013). The standardized model included *T*_air_, Treatment (i.e. humidity treatment), Sex, M_b_ and the *T*_air_:Treatment interaction with individual bird identity as a random fixed effect. The model was selected using Akaike weights as well as the Akaike information criterion values corrected for small sample sizes (AICc; Burnham and Anderson, 2002). Pseudoreplication was accounted for by including individual identity as a random factor in all models/analyses and significance was assessed using α < 0.05, with all values presented as mean ± SD. To test for significant heteroscedasticity between the *T*_b_, EWL and RMR data among humidity treatments or *T*_air_ categories, the *lmtest* package (Hothorn *et al.,* 2015) was used. As no deviations from normality or homoscedasticity were evident among residuals, using the *nlme* package (Pinheiro *et al*., 2022) we fitted a linear mixed effect model. To select the model with the highest explanatory power, the ‘model.sel’ function was again used in the same manner as above. For the response variables *T*_b_, RMR, EWL and EHL/MHP, the best models included *T*_air_, Treatment and *T*_air_:Treatment interaction; however, body mass and sex were not significant predictors and weakened the model and thus were excluded from further models. Finally, to compare maximum values of physiological variables (as well as the *T*_b_ and *T*_air_ values associated with the onset of panting) between humidity treatments, *post hoc* multiple comparisons Tukey HSD tests were used.

#### Partitioning air temperature and humidity contributions to lethal hyperthermia risk

We evaluated recent exposure to *T*_W_ ≥ *T*_W-max_ in our study area by using South African Weather Service *T*_air_ and relative humidity data for Richard’s Bay (S 28.737°, E 32.093°) in summer (October–March) from 2002 to 2022 to identify days on which hornbills experienced *T*_W_ ≥ *T*_W-max_. To estimate risks under future conditions, we applied increases in maximum *T*_air_ during December–February for the Richard’s Bay area projected for 2080–2100 relative to 1981–2010 for CMIP6 scenarios SSP2–4.5 and SSP5–8.5, obtained from the IPCC WGI Interactive Atlas (https://interactive-atlas.ipcc.ch/; IPCC, 2021). The increases in *T*_air_ for these two scenarios are 2.4 and 4.5°C, respectively. For projections of future humidity, we assumed that a) humidity increases with increasing *T*_air_ such that relative humidity in 2100 remains unchanged relative to current conditions, the scenario considered most likely for many regions (Sherwood and Meyer, 2006; Willett *et al.,* 2007). To partition the contributions of increases in *T*_air_ and increases in humidity to exposure to *T*_W_ ≥ *T*_W-max_ by 2100, we also modelled future exposure assuming absolute humidity remains unchanged, i.e. lower relative humidity by end-century.

#### Spatial variation in current and future exposure to lethal hyperthermia

We mapped recent (2021–23) and future (2080–2100) exposure to *T*_W_ ≥ *T*_W-max_ across the study species’ entire range. For recent simulations, we used the ERA5 global reanalysis climate model from Copernicus Climate Change Service implanted by the European Centre for Medium-Range Weather Forecasts. The ERA5 data are interpolated across a 31-km Gaussian grid, and the temporal coverage spans 1949 to 3 months prior to present day. To simulate daily future climate (i.e. *T*_air_ and humidity), we used experiment r1i1p and RCP scenarios 4.5 and 8.5 of the ACESS1–3 projection from CMIP5 (Copernicus Climate Change Service, Climate Data Store, 2021 DOI: 10.24381/cds.99842490, accessed on 15 June 2024). We selected these scenarios to represent a moderate- and high-risk future scenario, respectively. Climate models predicting the greatest rates of warming are also those that best simulate current conditions, suggesting that the unmitigated high-risk future climate change scenario (RCP 8.5) may be the most likely (Lee *et al.,* 2023). The future climate model uses forcing fields interpolated at 0.95° latitude × 1.25° longitude. Both the recent and future data were obtained from the Copernicus Climate Data Store (https://cds.climate.copernicus.eu/cdsapp#!/home), as part of a collaborative programme with strong model convergence. The temporal coverage was limited to daytime values (06 h00–18 h00) for both the recent and future simulations. Species distribution data were obtained from BirdLife International (http://datazone.birdlife.org/species/search). We also included a sensitivity analysis of spatial variation in exposure to *T*_W_ ≥ *T*_W-max_ if our predictions of *T*_W_ were under- or overestimated by 1 or 2°C.

#### Role of forest interior microclimates in buffering exposure to lethal hyperthermia

We evaluated the role of forest microclimates in reducing exposure to *T*_W_ ≥ *T*_W-max_ by comparing the predictions of microclimate models that consider vegetation structure to those based on macroclimate (i.e. only *T*_air_ and humidity). We used the *micro_ncep* function of the *NicheMapR* biophysical modelling packages (version 3.0; Kearney *et al.,* 2020) and the *microclima* package (Maclean *et al.,* 2019) in the R programming environment (R Core Team, 2019) using the R Studio (version 3.2.3) interface to predict microclimates. The microclimate model uses gridded climate data (~200 × 200 km) interpolated from the NOAA-NCEP reanalyses programme, and using digital elevation data and lapse corrections the model outputs are downscaled to resolutions between ~30 cm and 3 m (Maclean *et al.,* 2019; Kearney *et al.,* 2020). The outputs from *microclima* were used as input variables in the *microclimc* package (Maclean and Klinges, 2021), to predict within-canopy conditions using a steady-state heat transfer model. This model allows users to either manually specify vegetation and heat transfer parameters or use estimated values by specifying a habitat type. We used *microctools* to estimate vegetation characteristics (e.g. plant area index) and heat transfer (e.g. wind profiles) for each time period, location and height specified for the habitat type ‘mixed forests’. For the height category we assumed an average of 10 m above ground. A detailed description of the calculations, working of the parameters and their interactions is provided by Maclean and Klinges (2021). Based on satellite imagery we selected 10 × 10 km polygons within eight representative forested areas across the species’ entire range and extracted 10 random coordinates within each polygon to run the microclimate model. Model outputs per polygon were averaged to account for variation in leaf area index, topography, aspect, forest edge effects or elevation.

## Results

### Effects of humidity on thermoregulation

**Figure 1:**
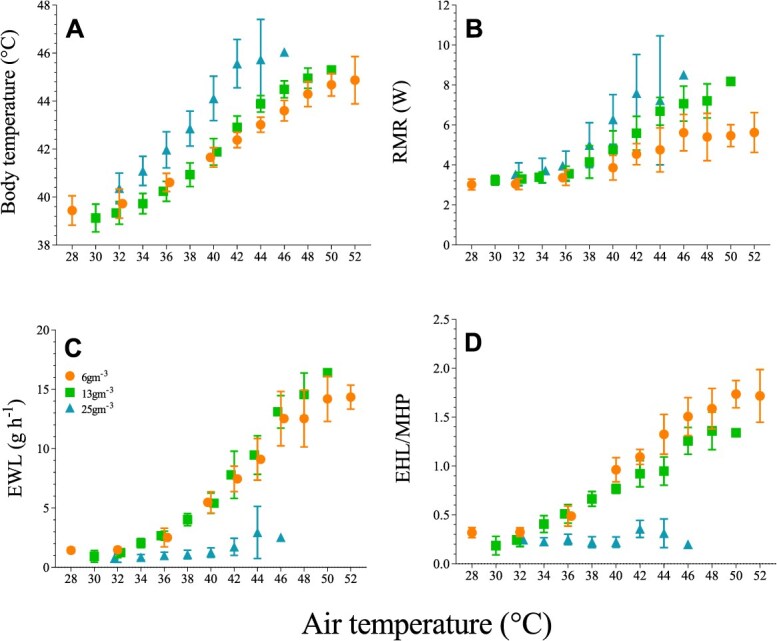
Relationships between air temperature (*T*_air_) and body temperature (*T*_b_, A), resting metabolic rate (B) and evaporative water loss (C) and evaporative heat loss (EHL)/metabolic heat production (MHP, D) in trumpeter hornbills (*B. bucinator*) experiencing experimental humidities of 6, 13 or 25 g m^−3^. Data are presented as means ± SD.

The heat tolerance and evaporative cooling capacity of *B. bucinator* decreased sharply with increasing humidity. Mixed effects models indicated maximum *T*_b_ (R^2^m = 0.73; R^2^c = 0.85) was significantly predicted by *T*_air_ (F_2,25_ = 25.71, *P* < 0.001), humidity treatment (F_2,25_ = 17.18, *P* < 0.001) and the *T*_air_ x humidity treatment interaction (F_2,25_ = 4.32, *P* < 0.001). The 25 g H_2_O m^−3^ treatment was associated with significantly higher maximum *T*_b_ (Tukey HSD = −1.01, *P* < 0.001) compared to the other two treatments. Mixed effects models (R^2^m = 0.83; R^2^c = 0.87) revealed that humidity treatment (*F*_2,25_ = 78.22, *P* < 0.001), *T*_air_ (*F*_2,25_ = 269.7, *P* < 0.001) and the *T*_air_ × humidity interaction (*F*_2,25_ = 3.48, *P* < 0.001) were significant predictors of heat tolerance limit, which was significantly lower in the 25 g H_2_O m^−3^ treatment (Tukey HSD = 7.55, *P* < 0.001) (Fig. 1A, Table S1, Table S2) by ~7.5°C compared to the 6 g H_2_O m^−3^ treatment. Whereas birds in both the low- and intermediate-humidity treatments were able to defend *T*_b_ below *T*_air_, individuals in the high-humidity treatment were not (Fig. 1A, Table S1).

These effects of high humidity on *T*_b_ and tolerance of high *T*_air_ were caused by significantly reduced evaporative cooling efficiency, for which *T*_air_ (*F*_2,25_ = 143.1, *P* < 0.001) and humidity treatment (*F*_2,25_ = 259.7, *P* < 0.001) emerged as significant predictors (Fig. 1D); at *T*_air_ = 44°C, rates of EWL in the high-humidity treatment were just 32.3 and 31.1% of those in the low and intermediate treatments. Reduced EWL, combined with a 52.1% increase in metabolic heat production compared to the low-humidity treatment, resulted in maximum EHL/MHP = 0.31 at *T*_air_ = 44°C in the high-humidity treatment, significantly lower (Tukey HSD = 1.14, *P* < 0.001, Fig. 1D) than the maxima of 1.74 at *T*_air_ = 50°C and 1.36 at *T*_air_ = 48°C achieved by hornbills in the low and intermediate treatments, respectively.

Heat dissipation behaviours during measurements also varied with humidity. During the high-humidity treatment, birds frequently tilted their heads upward, with copious saliva evident on the beak outer surfaces upon removal from the chamber (Fig. S1). The *T*_air_ associated with the onset of panting did not vary significantly among humidity treatments (F_2,25_ = 2.80, *P* = 0.079), but individuals experiencing humidity of 25 g H_2_O m^−3^ commenced panting at significantly higher *T*_b_ (Tukey HSD = −1.18, *P* = 0.002) compared to the other two treatments (Table S1).

### Maximum wet-bulb temperature

Hornbills in the high-humidity treatment showed loss of coordination and balance at *T*_W_ = 31.7 ± 1.0°C (range = 30.4–33.1°C). This value meets our criteria for ecologically relevant avian *T*_W-max_, as hornbills had reached thermal endpoints and were incapable of maintaining *T*_b_ below environmental temperature, which in the metabolic chamber can be assumed to equal *T*_air_.

### Partitioning air temperature and humidity contributions to lethal hyperthermia risk

During the period 2002–22, *T*_W_ calculated from weather station *T*_air_ and humidity in the Richards Bay area exceeded the hornbills’ *T*_W-max_ = 31.7°C on an average of 0.95 days year^−1^. By the end of the century, exposure will increase to 3.5 days year^−1^ under the SSP2–4.5 scenario and to 13.8 days year^−1^ under the SSP5–8.5 scenario. Under the SSP2–4.5 scenario, 39% of the increased exposure to days with *T*_W_ > 31.7°C is attributable to increases in *T*_air_ and the remaining 61% to increases in humidity. The proportional contribution of increased humidity under the SSP5–8.5 scenario, however, is 87%, with projected increases in exposure of just 1.9 days year^−1^ if absolute humidity were to remain at current levels.

### Spatial variation in current and future exposure to lethal hyperthermia

**Figure 2:**
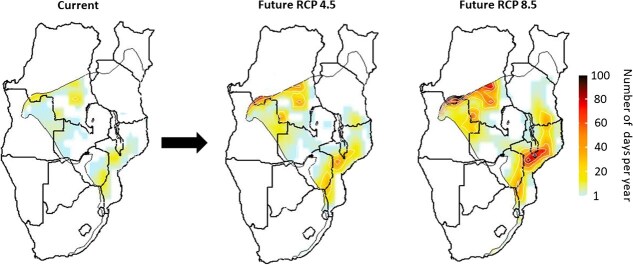
Exposure of trumpeter hornbills (*B. bucinator*) to wet-bulb temperature (*T*_W_) exceeding the species-specific critical *T*_W_ of 31.7°C under recent conditions (2021–23, left panel) and under climates projected for 2080–2100 using an RCP 4.5 (centre) or RCP 8.5 scenario (right).

Assuming no microclimate buffering, under recent climate, hornbills are exposed to *T*_W_ ≥ *T*_W-max_ on at least 1 day year^−1^ over ~30% of their range, with exposure highest in southern and central Mozambique and the southern Democratic Republic of Congo (DRC; Fig. 2). By the end of the century, exposure will increase 6-fold in central Mozambique and 16-fold in southern DRC, with exposure as high as 82–100 days year^−1^ in these two regions (Fig. 2). Exposure on at least 1 day year^−1^ will occur over ~46% of the species’ current range. The sensitivity analysis revealed that our projections are sensitive to species-specific *T*_w-max_, with overestimation or underestimation by 1–2°C associated with large changes in modelled exposure under both RCP 8.5 (Fig. S2) and RCP 4.5 (Fig. S3).

### Role of forest interior microclimates in buffering exposure to lethal hyperthermia

**Figure 3:**
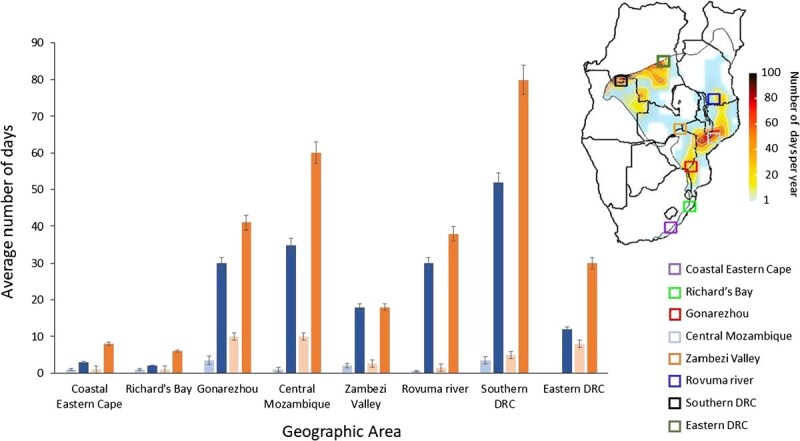
Forest microclimates reduce exposure of trumpeter hornbills (*B. bucinator*) to lethal hyperthermia risk, with the number of days under present climate (lighter bars) and climate projected for 2080–2100 under an RCP 8.5 scenario (darker bars). The blue bars indicate values predicted by a microclimate model that considers vegetation structure and the consequent shading, whereas the orange bars indicate values predicted on the basis of macroclimate (i.e. *T*_air_ and humidity alone). Current and future exposure is shown for eight areas in the species’ range.

Our microclimate model generally predicted lower exposure to *T*_W_ ≥ *T*_W-max_ compared to the macroclimate model (Fig. 3), supporting the prediction that cool, shaded microclimates within forest interiors reduce the risk of lethal hyperthermia experienced by birds. Across the modelled sites, the microclimate model predicted exposure equivalent to ~33.3–99.7% of values predicted by the macroclimate model (Fig. 3).

## Discussion

The prediction that increases in humidity will result in substantially greater risks of lethal hyperthermia for birds inhabiting humid lowland habitats was supported by our empirical data and models of exposure under recent and anticipated future climate. For our study species, exposure to lethal hyperthermia associated with conditions when *T*_W_ exceeds *T*_W-max_ will increase substantially by 2100 under a business-as-usual emissions pathway, assuming relative humidity remains constant. These increases primarily reflect rising humidity rather than higher *T*_air_*per se*. Our models also support the prediction that exposure will be reduced for birds occupying shaded microsites in forest interiors, providing a quantitative example of the importance of intact forests for reducing birds’ exposure to potentially lethal conditions. As we discuss below, our findings suggest rising temperatures will severely impact birds occupying humid lowlands at subtropical and tropical latitudes and emphasize the need to incorporate humidity in models of impacts of climate change on global avian biodiversity.

Our data reveal an upper wet-bulb temperature limit of ~32°C for an avian forest frugivore inhabiting tropical and subtropical latitudes. The *T*_W-max_ of 31.7°C we estimate here for a forest bird is ~3°C below the critical *T*_W_ of 35°C proposed for resting humans (Sherwood and Huber, 2010,), and similar to the values of 30–31°C recently reported for humans engaged in light exercise intended to simulate activity levels typical of daily living in warm, humid environments (Vecellio *et al.,* 2022). The absence of comparable avian *T*_W-max_ values precludes direct comparisons with other bird species. Our modelling approach assumes birds are resting in complete shade and are shielded from direct or reflected solar radiation, i.e. operative temperature (*T*_e_, Bakken, 1976; Robinson *et al.,* 1976) is equal to *T*_air_. Our predictions should therefore be considered conservative estimates of exposure to *T*_W_ ≥ *T*_W-max_, as neither external heat loads associated with partially shaded microsites nor internal heat loads generated by activity or digestion are taken into account. Although variation of 1–2°C in estimated *T*_W-max_ has a large effect projected exposure, the measurement errors in *T*_air_ or humidity necessary to over- or underestimate *T*_w-max_ by this magnitude are large. A difference of 2°C between measured and actual *T*_w-max_ would require measurement errors of ~7°C for *T*_air_ or ~20% for absolute humidity. Moreover, our approach follows that of several recent studies of human exposure to deadly conditions based on threshold values of *T*_W_ (Sherwood and Huber, 2010; Freychet *et al.,* 2022; Vecellio *et al.,* 2023).

As expected, high humidity reduced the thermoregulatory performance of hornbills by impeding evaporative heat dissipation, evident as increases in EWL in the high-humidity treatment equivalent to only 23% of those achieved under low humidity. These decreases in maximum EWL with increasing humidity are qualitatively consistent with those observed in birds from several habitat types (Gerson *et al.,* 2014; Freeman *et al.,* 2024). The excessive saliva production we observed in hornbills at high humidity, to the point that saliva was dripping off their beaks, supports arguments that beak-wetting can contribute to avian heat dissipation (Bartholomew and Cade, 1957; Janse *et al.,* 2020; Zuluaga and Danner, 2023). The possibility that the fluid on the hornbills’ beaks occurred through condensation can be ruled out by the chamber dew point in the high-humidity treatment being ~27°C, well below the experimental *T*_air_ range.

We investigated the thermal physiology of a single species, but data for other forest-dwelling birds suggest quantitatively similar effects of high humidity on thermoregulation. In particular, the maximum *T*_b_ (46.6°C) of the hornbills at an experimental humidity of 25 g H_2_O m^−3^ is similar to the mean of 46.3 ± 0.5°C at 19 g H_2_O m^−3^ reported for 13 southern African species occupying humid lowland forest habitats (Freeman *et al.,* 2024). The chamber *T*_W_ values at which these *T*_b_ maxima were reached in Freeman *et al.*’s (2024) study were equivalent to 30.0 ± 0.4°C, but as maximum EHL/MHP remained >1.0 for most species these *T*_W_ values do not meet our criteria for *T*_W-max_. We consider it unlikely that *T*_W-max_ for any of these species is much higher than that of the hornbills, although further research is necessary to confirm this.

The large increases in exposure to potentially lethal conditions under future climates we report here have implications for understanding global patterns of avian vulnerability to warming. Many analyses of the vulnerability of birds inhabiting lowland forests to climate change have focused on expected range shifts of food resources such as fruiting plants and insects towards higher elevations and latitudes (e.g. Şekercioğlu *et al.,* 2012; Freeman *et al.,* 2018a) and interactions with the impacts of land use change, habitat degradation and other anthropogenic effects (Jetz *et al.,* 2007; Sherry, 2021). Far less attention has focused on physiological constraints and direct impacts of changing thermal environments. On the basis of differences between local maximum *T*_air_ and species’ heat tolerance limits and upper critical limits of thermoneutrality for 58 tropical bird species at 9°N in Panama and 23 species at 33°N in South Carolina, (Pollock *et al.,* 2021) concluded that tropical species are not systematically more vulnerable to rising *T*_air_ than species from temperate latitudes. Our findings here, however, suggest the risk of lethal hyperthermia will expand greatly for tropical birds inhabiting warm, humid regions, and reveal a direct mechanism whereby populations may decline on the warm edge of species’ ranges. For example, range expansions by lowland species that have rapidly colonized upslope areas in Peru (Freeman *et al.,* 2018a) may be offset in coming decades by the effects of increasing humidity in warmer parts of their ranges.

Several analyses of the risks of lethal dehydration or hyperthermia for birds in arid (McKechnie and Wolf, 2010; Albright *et al.,* 2017; Conradie *et al.,* 2020) and Mediterranean (Cabello-Vergel *et al.,* 2022) climates have shown that mortality risk during extreme heat events will increase by 2100. Our analysis of the end-century exposure of a tropical frugivore to *T*_W_ > *T*_W-max_ shows that birds in mesic, humid regions will experience similar increases in mortality risk during more intense and frequent heatwaves. This finding contrasts with the suggestion that subtropical arid regions, but not tropical forests, are hotspots of avian physiological vulnerability to climate change (Monge *et al.,* 2023), but is consistent with arguments that humid conditions increase the likelihood of mass mortality events among birds and bats (Welbergen *et al.,* 2008; Ratnayake *et al.,* 2019; McKechnie *et al.,* 2021b). Moreover, our findings contradict the idea that effects of warming on tropical species will primarily occur via indirect effects rather than direct physiological impacts (Freeman *et al.,* 2018a,), or that direct effects of warming will be less important for endothermic compared to ectothermic vertebrates (Aragón *et al.,* 2010).

Our analysis of the buffering effect of forest vegetation on exposure to *T*_W_ ≥ *T*_W-max_ reiterates that the vulnerability of forest species to increasing *T*_air_ and more frequent heat events is exacerbated by reductions in natural vegetation via anthropogenic transformation and the loss of structurally complex vegetation (Fahrig *et al.,* 2011; Olivier *et al.,* 2013; Rolo *et al.,* 2017). Forest fragmentation and degradation results in the loss of cool interior microsites and exposure to higher *T*_air_ and greater solar heat loads (De Frenne *et al.,* 2021), changes linked to declines in abundance in birds occupying temperate forests (Kim *et al.,* 2022). Overall, our findings here emphasize the potential role of thermal environments experienced by forest birds as a determinant of species-area relationships in fragmented forest landscapes (e.g. Freeman *et al.,* 2018b).

We focused on wet-bulb temperature, which incorporates *T*_air_ and humidity but not solar radiation or forced convection. For this reason, our approach is appropriate for birds in the shaded interior of forests, but likely underestimates exposure for species occupying exposed, sunlit microsites and which experience higher environmental temperatures on account of solar heat gain. For species occupying exposed microsites, thermal indices such as wet-bulb globe temperature (WBGT) incorporating solar radiation may provide more accurate risk estimates (Cooper and Withers, 2024; Mason *et al.,* 2024), but the applicability of WBGT and other indices of thermal comfort parameterized for humans to other taxa may be limited. We also emphasize that we have modelled only acute, lethal risks of hot weather in this analysis. Sublethal fitness costs of hot weather, reflecting missed opportunities arising from behavioural trade-offs between foraging and thermoregulation (Cunningham *et al.,* 2021), pose a major threat to arid-zone birds in the face of rapid warming (Conradie *et al.,* 2019; Pattinson *et al.,* 2022). For birds in humid lowland habitats, these trade-offs are likely exacerbated by reduced evaporative cooling efficiency during periods of elevated humidity. We anticipate that sublethal costs associated with chronic exposure to sustained hot weather, which includes reduced body condition (du Plessis *et al.,* 2012; Sharpe *et al.,* 2019; van de Ven *et al.,* 2019) and compromised breeding success (Cunningham *et al.,* 2013; Wiley and Ridley, 2016; van de Ven *et al.,* 2020), are greater during periods when high *T*_air_ coincides with high humidity.

In conclusion, our analysis of recent and future exposure of an avian forest frugivore to combinations of *T*_air_ and humidity exceeding thermoregulatory limits reveals that hyperthermia risk will increase substantially in coming decades. Tropical forests are often considered climatically benign and thermally unchallenging for birds, with indirect impacts of rising temperatures thought to be the main determinants of avian vulnerability to climate change in these habitats. However, our findings suggest increasing *T*_air_ and concomitant increases in humidity will result in more frequent exposure to thermal conditions resulting in lethal hyperthermia. The thermal challenges associated with hot, humid conditions are important to consider in analyses of the effects of changing precipitation regimes and the hygric niches (Boyle *et al.,* 2020) occupied by organisms. Longer dry seasons in many tropical regions are anticipated to drive species losses and changes in tropical bird community composition under business-as-usual emission scenarios (Brawn *et al.,* 2017, 2024). However, direct mortality and sublethal fitness costs arising from temperature- and humidity-dependent behavioural trade-offs are likely to increase during wet seasons, even if they are shorter than in the past. Recently documented avifaunal declines in undisturbed tropical rainforests (Stouffer *et al.,* 2021; Wolfe *et al.,* 2025) underscore the need for a better understanding of physiological aspects of species’ vulnerability to climate change.

## Supplementary Material

Web_Material_coaf036

## Data Availability

The data collected in this study are available at https://data.mendeley.com/datasets/d8r9sz6yyz/1
