# Supplementary material for: Increases in humidity will intensify lethal hyperthermia risk for birds occupying humid lowlands
Source: Conserv Physiol. 2025 Jun 3;13(1):coaf036. doi: 10.1093/conphys/coaf036 (PMC12133222; doi:10.1093/conphys/coaf036)
Supplement: Web_Material_coaf036 [file web_material_coaf036.zip › Coulson et al supplementary materials.pdf]

Supplementary materials

Increases in humidity will intensify lethal hyperthermia risk for birds occupying humid lowlands

Bianca Coulson
Marc T. Freeman
Shannon R. Conradie
Andrew E. McKechnie

Supplementary Methods

S.M.1 *Air and body temperature measurements*

Prior to experiments, a temperature-sensitive passive integrated transponder (PIT) tag (Biotherm 13, Biomark, Boise, ID, USA) was injected subcutaneously into the abdominal cavity of each hornbill for body temperature (T_b) measurements following Czenze et al. (2020) and Freeman et al. (2022; 2024). Two reader-transceiver systems (HPR+, Biomark, Boise ID, USA) placed against the chamber were used to read T_b values from the PIT tags. All PIT tags were calibrated at temperatures of 30-50°C in a circulating water bath (model F34, Julabo, Seelbach BW, DE) against a thermocouple meter (TC-1000, Sable Systems, Las Vegas, NV, USA) and a mercury-in-glass thermometer with NIST-traceable accuracy. Temperatures measured using the PIT tags deviated from actual values by 0.07 ± 0.08 °C (n = 15), with values corrected prior to analysis. A thermistor probe (TC-100, Sable Systems, Las Vegas, NV, USA) was used to measure T_{air} in the chamber during gas exchange measurements. The probe was inserted through a small hole in one side of the respirometry chamber and sealed using a rubber grommet.

S.M.2 *Gas exchange measurements*

We used an open flow-through respirometry system similar to that described by Freeman et al. (2022; 2024) to measure evaporative water loss (EWL) and carbon dioxide production ($\dot{V}_{CO_2}$). Birds were individually placed in a sealed 30-L chamber (620mm long x 395mm high x 320mm wide) constructed from transparent polycarbonate, fitted with a mesh platform 10 cm above a

~1-2 cm mineral oil layer to prevent evaporation from excreta from affecting evaporative water loss (EWL) measurements. The metabolic chamber was placed within a custom-built thermally insulated controlled-temperature unit (1m high x 1.2m wide x 1m deep) where T_{air} inside was regulated by a Peltier device (AC-162 Thermoelectric Air Cooler, TE Technology, Traverse City MI, USA) controlled using a digital controller (TC-36–25-RS485 Temperature Controller, TE Technology, Traverse City MI, USA).

For all treatments, baseline and chamber air were subsampled in manual mode through a respirometry multiplexer (model MUX3-1101-18M, Sable Systems) at a flow rate of ~160 mL min^{-1} regulated by a subsampling pump (model SS4, Sable Systems, Las Vegas NV, USA) and further pulled through a LI-COR, i.e., CO_2/H_2O analyser (LI-840A, LI-COR, Lincoln NE, USA) followed by an O_2 analyser (FC-10A, Sable Systems, Las Vegas NV, USA). The LI-COR was regularly zeroed using pure nitrogen (AFROX, Johannesburg, South Africa), and spanned using a 2000 ppm CO_2 in N_2 gas mix (AFROX) and humidified air with a dewpoint 5 – 6 °C below ambient T_{air} generated using a dew point generator (DG-4, Sable Systems, Las Vegas NV, USA). The O_2 analyser was used to measure barometric pressure and was periodically spanned to 20.91% using dry air.

During measurements, data were acquired every 5 s using an analog-digital converter (model UI-3, Sable Systems, Las Vegas NV, USA) which converted voltage inputs to digital values. The values from the UI-3 were then recorded using Expedata software (Sable Systems, Las Vegas NV, USA) on a computer.

S.M.3 *Experimental manipulation of chamber humidity*

Humidity in the respirometry chamber was regulated following Freeman et al., (2024) at one of three humidity levels: ~6, 13 or 25 g H_2O m^{-3}, hereafter referred to as low, intermediate and high humidity treatments, respectively. Under natural conditions, trumpeter hornbills in our study area experience absolute humidities ranging from 6 g H_2O m^{-3} to 37 g H_2O m^{-3}, however, given the practicalities of manipulating in-chamber humidity while ensuring no condensation in the respirometry system, 25 g H_2O m^{-3} was the highest feasible experimental humidity. Briefly, atmospheric air was supplied to the chamber via an oil-free compressor and dried using a membrane drier (Atlas Copco SD1N air dryer and filter, Atlas Copco, Stockholm, Sweden). Downstream of the compressor and membrane drier, the air stream was split into two channels (baseline and experimental) using Bev-A-Line IV tubing (Thermoplastic Processes Inc., Warren, NJ, USA). To regulate flow rate to the baseline channel ("dry baseline"), a needle

valve (Swagelok, Solon, OH, USA) was used and kept the flow rate at ~ 1 L min^{-1}. For the low humidity treatment (~6 g H_2O m^{-3}), the incurrent channel that provided air to the metabolic chamber was split into two lines, each with a mass flow controller (MFC) (30 SLPM or 50 SLPM, Alicat Scientific Inc., Tuscon AZ, USA), downstream of which they re-joined before entering the chamber. Flow rates were adjusted to maintain humidity within the metabolic chamber at the desired set point and varied between 15 L min^{-1} and 80 L min^{-1}.

For the intermediate and high humidity treatments, the experimental line was also split again into two channels. The first channel's ("humid stream") flow rates were regulated using an MFC (20 SLPM, Alicat Scientific Inc., Tuscon AZ, USA) at rates of 1 – 4 L min^{-1} before the air passed through three water-filled bubblers connected in series. Each bubbler consisted of a 3-L screw-top, sealable bottle (diameter = 14 cm, height = 25 cm; Universal Jar, Tupperware, Orlando, FL, USA) fitted with in- and outlet fittings. These fittings were installed on the lid and incurrent air passed through the tubing and an aquarium stone ~1 cm above the bottom of the water column. The first of the three in-series bubblers was kept at T_{air} = ~35 °C, whereas the remaining two were kept at a constant T_{air} slightly above the desired chamber dewpoint (~16°C and ~27°C respectively) in a temperature-controlled chamber (PELT-5, Sable Systems, Las Vegas NV, USA).

The second experimental channel consisted of dry air with flow rates controlled by another MFC (10 SLPM, Alicat Scientific Inc., Tuscon AZ, USA) that merged with the humid stream downstream of the bubblers. Mixing humidified and dry air provided more precise control of humidity within the chamber compared to when only humidified air from the bubblers was used (Freeman et al., 2024). By regularly adjusting dry air flow rates, it was possible to precisely regulate humidity levels in the chamber despite EWL increasing with T_{air}. Downstream from the humid/dry merge, the channel was split into incurrent baseline channel with flow rate regulated by a needle valve (~1 L min^{-1}) to allow for measurement of incurrent humidity. Downstream of this final split, incurrent flow rates were measured before the chamber inlet using a MFC (20 SLPM, Alicat Scientific Inc., Tuscon AZ, USA) set to its maximum flow rate and allowed to act as a mass flow meter at flow rates < 20 L min^{-1}. All MFCs were calibrated using a Gilibrator 2 (Sensidyne, St Petersburg, FL, USA). Incurrent flow rates varied between 7.0 – 17.4 L min^{-1} for the intermediate and 1.4 – 7.3 L min^{-1} for the high humidity treatment. The washout time expected for the system to reach 99% equilibrium at the intermediate treatment, given a volume of 30 L and an average flow rate of 9.6 ± 2.5 L min^{-1}, was ~15 min (Lasiewski et al., 1966) while the high treatment (average flow rate = 4.8 ± 1.6 L

min $^{-1}$) was ~30 min. Therefore, to ensure that the chamber had reached equilibrium of both humidity and T_{air} after adjusting flow-rates, we implemented transitional periods of ~30-35 min before measurements between each set T_{air}. If humidity values or air temperature were still transitioning or were unstable, additional time would be given to allow data to be collected from stable traces at desired humidity levels.

Absolute humidities within the metabolic chambers were 5.56 ± 0.26 g H_2O m^{-3} (n = 10) for the low treatment, 13.36 ± 0.47 g H_2O m^{-3} (n = 10) for the intermediate treatment and 24.51 ± 0.49 g H_2O m^{-3} (n = 8) for the high treatment. These humidities, dewpoints of 4°C, 16°C and 27°C, respectively, are equivalent to those often experienced by birds in lowland, coastal KwaZulu Natal [also see humidities used in other studies: (Gerson et al., 2014; Powers, 1992; van Dyk et al., 2019; Freeman et al., 2024)]. To avoid condensation in analysers and tubing, particularly during measurements at the high humidity treatment, T_{air} in the room where measurements took place was maintained at ~ 35-40 °C.

S.M Table 1. Summary of thermoregulatory performance as a function of chamber air temperature (T_{air}) at humidities of ~6, 13 and 25 g H_2O m^{-3}, respectively, of Trumpeter hornbills (*Bycanistes bucinator*) from a humid lowland study site in eastern South Africa. T_b = body temperature, T_a = ambient temperature, RMR = resting metabolic rate, EWL = evaporative water loss, EHL= evaporative heat loss, MHP = metabolic heat production. Values are presented as means ± SD, with sample sizes in parentheses.

	**Humidity treatment**		
Variable	**6 g m^{-3}**	**13 g m^{-3}**	**25 g m^{-3}**
**Body mass (g)**	703.7 ± 83.4 (10)	676.5 ± 109.1 (10)	666.9 ± 116.8 (8)
**Body temperature**			
Min. T_b (°C)	38.15 ± 0.81 (10)	37.60 ± 0.77 (10)	39.4 ± 0.64 (7)
Inflection T_{air} (°C)	33.87	------	------
T_b versus T_{air} slope (T_b °C T_{air} °C^{-1})	0.31	0.42	0.67
Max T_b (°C)	45.40 ± 0.55 (10)	45.67 ± 0.24 (10)	46.55 ± 0.40 (8)
Max T_{air} (°C)	51.07 ± 1.17 (10)	48.89 ± 0.89 (10)	43.66 ± 1.87 (8)
T_b at onset of panting (°C)	40.83 ± 0.69 (10)	40.11 ± 0.54 (10)	41.29 ± 0.60 (8)
T_{air} at onset of panting (°C)	36.32 ± 1.87 (10)	35.91 ± 1.41 (10)	34.38 ± 2.11 (8)
**Metabolic rate**			
Min. RMR (W)	2.34 ± 0.83 (10)	2.58 ± 1.07 (10)	2.60 ± 0.64 (8)
T_{uc} (°C)	35.41	35.77	------
RMR slope (mW °C^{-1})	186.15	347.69	582.76
Max. RMR (W)	7.90 ± 1.39 (10)	9.25 ± 1.16 (10)	12.03 ± 2.17 (8)
Max. RMR/min. RMR	3.38	3.59	4.63
**Evaporative water loss**			
Min. EWL (g h^{-1})	0.85 ± 0.31 (10)	0.92 ± 0.68 (10)	0.76 ± 0.36 (8)
Inflection T_{air} (°C)	37.26	------	------
EWL slope (g h^{-1} °C^{-1})	0.92	1.16	0.22
Max. EWL (g h^{-1})	14.34 ± 0.55 (8)	14.56 ± 1.73 (6)	2.94 ± 0.88 (3)
Max. EWL/min. EWL	16.87	15.82	3.86
Min. EHL/MHP	0.20 ± 0.46 (10)	0.06 ± 0.13 (10)	0.05 ± 0.06 (8)
EHL/MHP inflection T_{air} - T_b (°C)	-4.78	------	------
EHL/MHP slope	0.12	0.11	0.01
Max. EHL/MHP	1.94 ± 0.59 (10)	1.58 ± 0.18 (10)	0.36 ± 0.06 (8)

S.M Table 2 Tukey HSD post-hoc test comparing maximum body temperatures (T_b*max*), normothermic body temperatures (T_b*norm*) at 32°C, rate of body temperature change ($T_b$slope) heat tolerance limits (HTL), maximum rates of evaporative water loss (EWL), maximum rates of resting metabolic rate (RMR), maximum evaporative heat loss(EHL)/ metabolic heat production (MHP) as well as the T_b and T_{air} values for the onset of panting for *B.bucinator* exposed to increasing T_{air} at three different absolute humidity treatments (6, 13 and 25 g m^{-3}). Significant difference is indicated by bold p-values ($p<0.05$). Upper and lower confidence limits (95%) of the honest significant difference (HSD) are provided.

	HSD	95% CI lower	95% CI upper	p-value
T T_b***max***				
6gm^{-3} vs. 13gm^{-3}	-0.24	-0.78	0.29	0.511
6gm^{-3} vs. 25gm^{-3}	-1.26	-1.82	-0.70	**<0.001**
13gm^{-3} vs. 25gm^{-3}	-1.01	-1.58	-0.46	**<0.001**
T_b***norm* at** T_{air} **= 32°C**				
6gm^{-3} vs. 13gm^{-3}	-1.02	-1.94	-0.11	**0.026**
6gm^{-3} vs. 25gm^{-3}	-0.64	-1.55	0.27	0.207
13gm^{-3} vs. 25gm^{-3}	0.39	-0.44	1.21	0.487
**HTL**				
6gm^{-3} vs. 13gm^{-3}	2.18	0.74	3.62	**0.002**
6gm^{-3} vs. 25gm^{-3}	7.55	6.02	9.07	**<0.001**
13gm^{-3} vs. 25gm^{-3}	5.35	3.84	6.89	**<0.001**
**EWL**				
6gm^{-3} vs. 13gm^{-3}	0.99	-1.09	3.08	0.472
6gm^{-3} vs. 25gm^{-3}	13.63	11.42	15.84	**<0.001**
13gm^{-3} vs. 25gm^{-3}	12.64	10.42	14.85	**<0.001**
**RMR**				
6gm^{-3} vs. 13gm^{-3}	-1.19	-2.82	0.44	0.180
6gm^{-3} vs. 25gm^{-3}	-1.71	-3.44	0.01	**0.049**
13gm^{-3} vs. 25gm^{-3}	-0.53	-2.26	1.20	0.731
**EHL/MHP**				
6gm^{-3} vs. 13gm^{-3}	0.44	0.27	0.60	0.209
6gm^{-3} vs. 25gm^{-3}	1.57	1.40	1.75	**<0.001**
13gm^{-3} vs. 25gm^{-3}	1.14	0.96	1.31	**<0.001**
T_b **at onset of panting**				
6gm^{-3} vs. 13gm^{-3}	0.72	-0.003	1.44	0.051
6gm^{-3} vs. 25gm^{-3}	-0.46	-1.23	0.31	0.320
13gm^{-3} vs. 25gm^{-3}	-1.18	-1.95	-0.41	**0.002**
T_{air} **at onset of panting**				
6gm^{-3} vs. 13gm^{-3}	0.41	-1.71	2.53	0.878
6gm^{-3} vs. 25gm^{-3}	1.94	-0.31	4.19	0.102
13gm^{-3} vs. 25gm^{-3}	1.53	-0.72	3.77	0.231

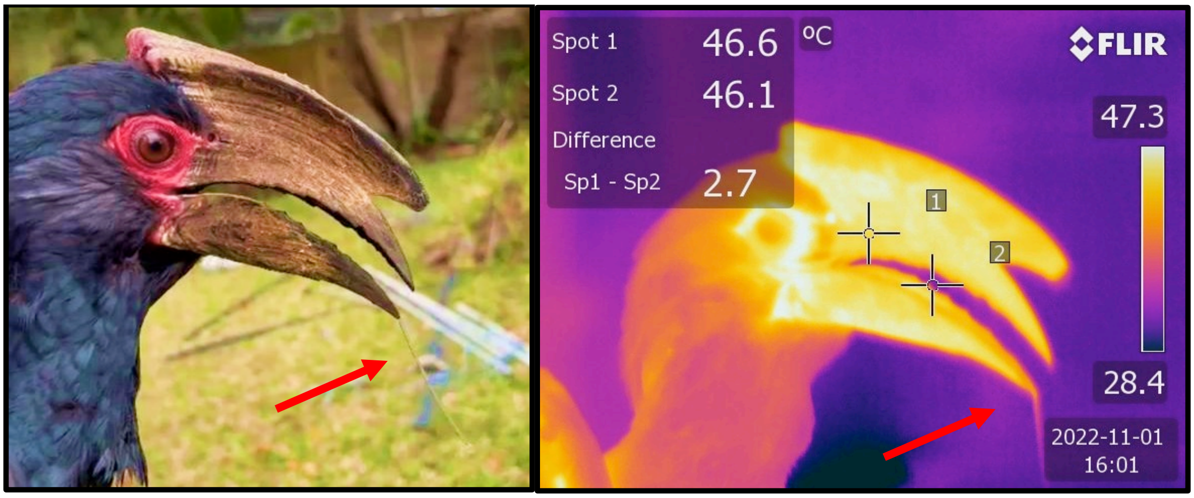

S.M Figure 1: Trumpeter hornbill (*Bycanistes bucinator*) producing excessive saliva after exposure to raised humidity and high air temperatures (~42 - 44°C).

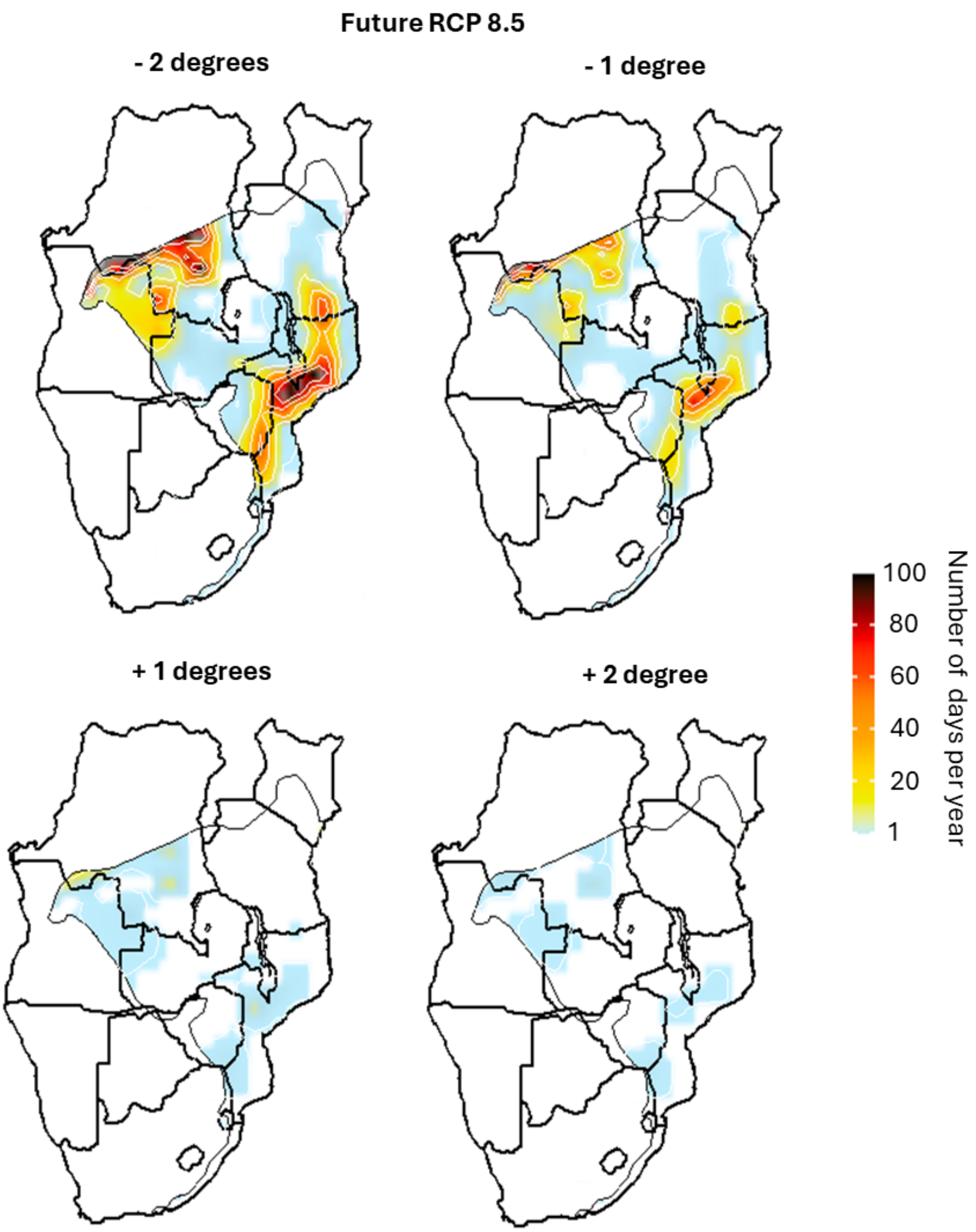

S.M Figure 2. Exposure of trumpeter hornbills (*Bycanistes bucinator*) to wet bulb temperatures (T_W) exceeding the empirically-determined species-specific maximum T_W of 31.7 °C if this value was underestimated (upper panels) or overestimated (lower panels) by 1 °C (left panels) or 2 °C (right panels) under climates projected for 2080-2100 using an RCP 8.5 scenario.

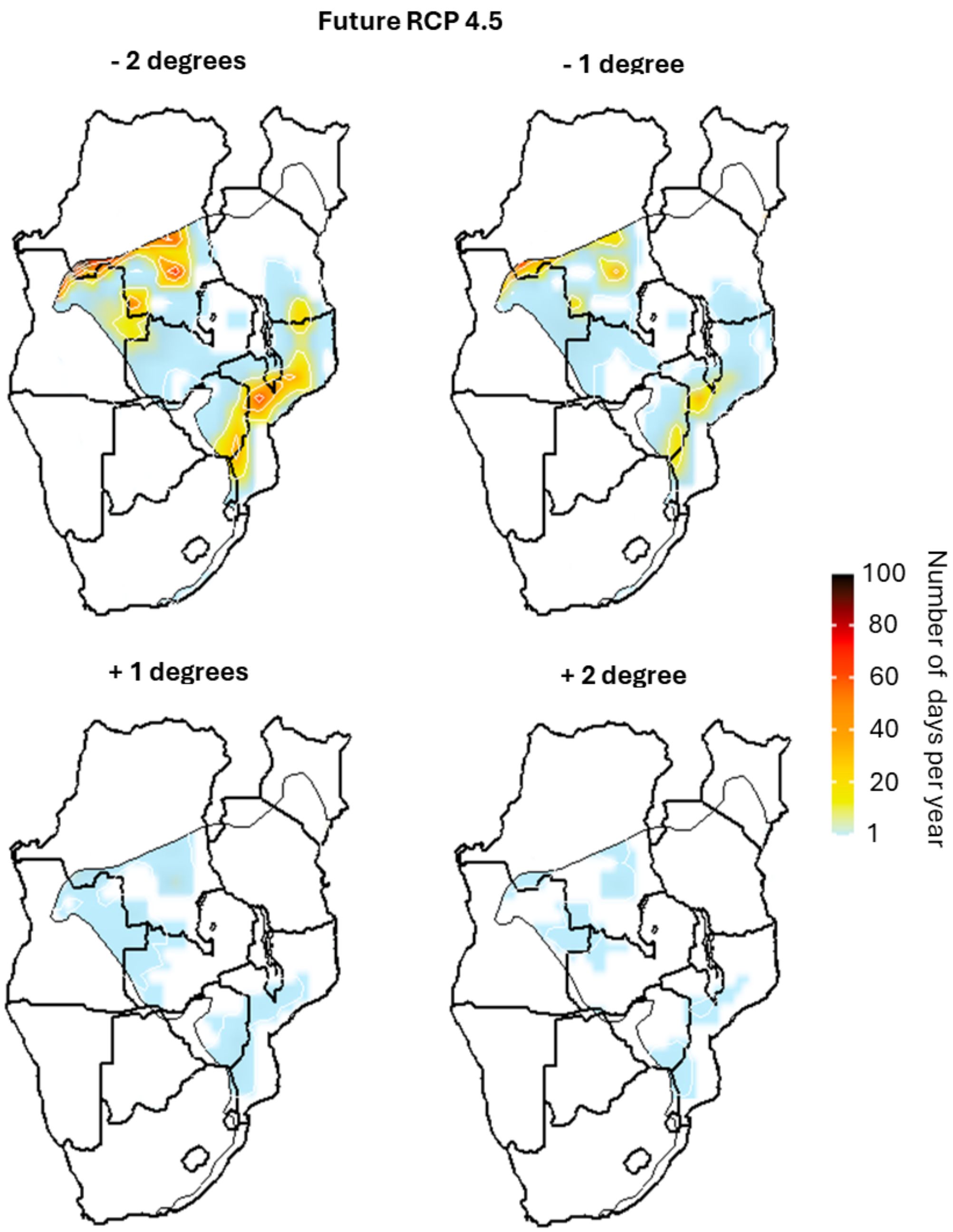

S.M. Figure 3. Exposure of trumpeter hornbills (*Bycanistes bucinator*) to wet bulb temperatures (T_W) exceeding the empirically-determined species-specific maximum T_W of 31.7 °C if this value was underestimated (upper panels) or overestimated (lower panels) by 1 °C (left panels) or 2 °C (right panels) under climates projected for 2080-2100 using an RCP 4.5 scenario.
