# Supplementary material for: Increases in humidity will intensify lethal hyperthermia risk for birds occupying humid lowlands
Source: Conserv Physiol. 2025 Jun 3;13(1):coaf036. doi: 10.1093/conphys/coaf036 (PMC12133222; doi:10.1093/conphys/coaf036)
Supplement: Web_Material_coaf036 [file web_material_coaf036.zip › Graphical abstract.pdf]

How will the combined effects of increasing air temperature and humidity affect risks of lethal hyperthermia for a forest frugivore?

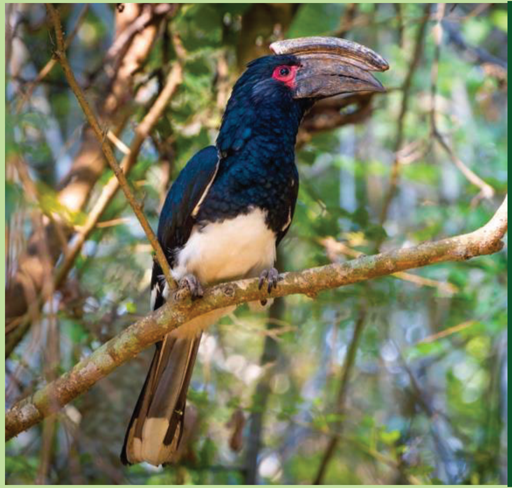

Trumpeter hornbills (*Bycanistes bucinator*) occupy forested habitats in sub-Saharan Africa.

The maximum wet bulb temperature tolerated by hornbills under resting conditions was 31.7 ± 1.0 ºC.

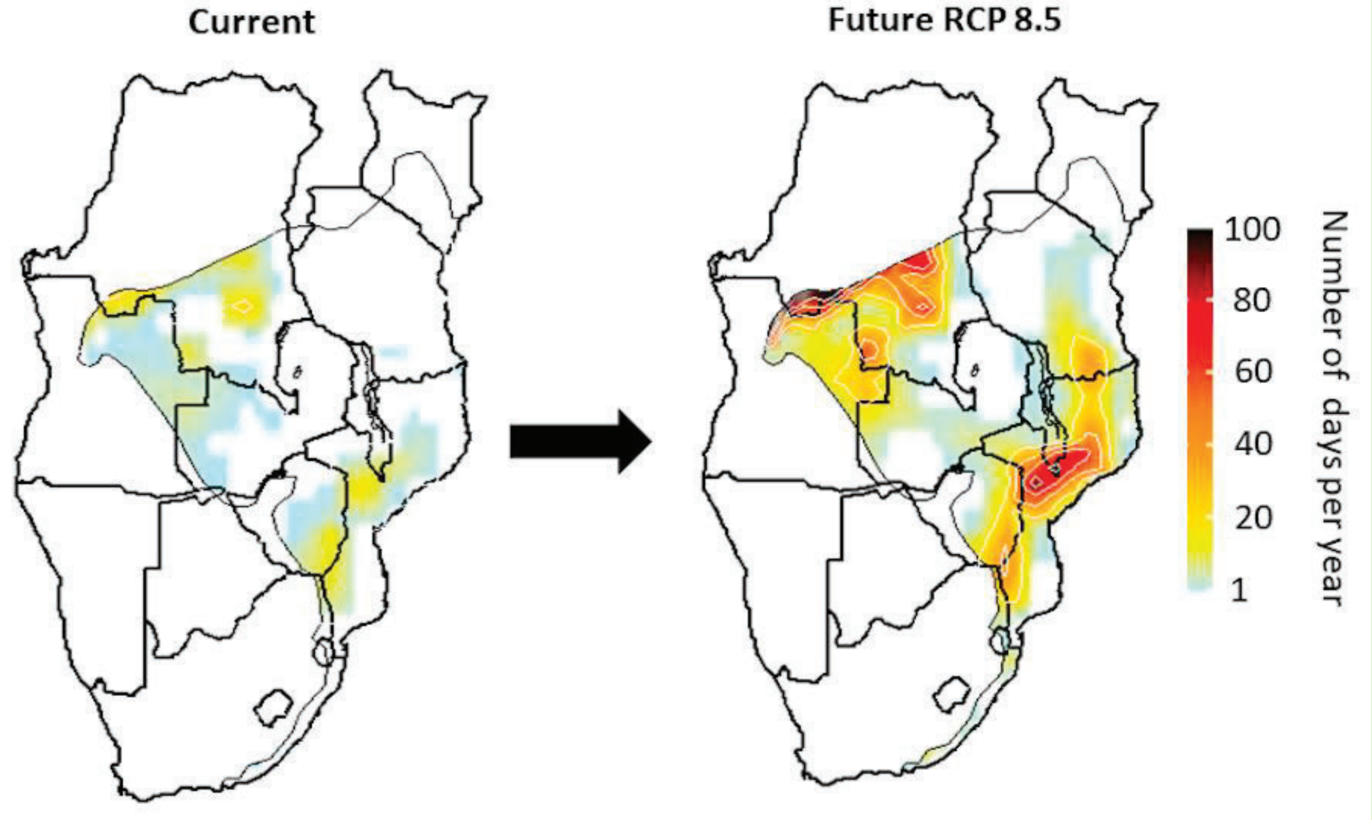

Exposure of trumpeter hornbills to wet bulb temperatures exceeding 31.7 ºC under current climate (left) and the period 2080 – 2100 (right)

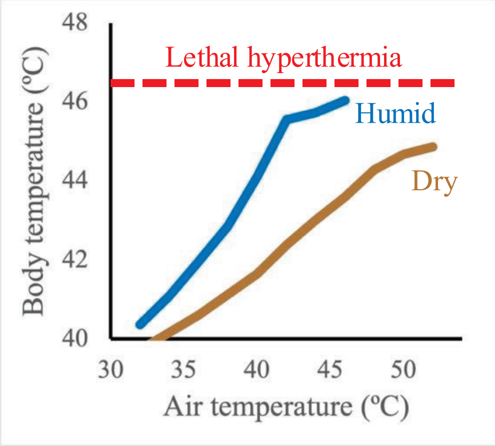

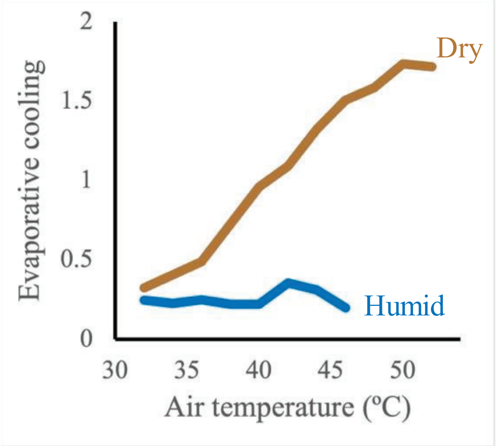

Body temperature (left) approached lethal limits more rapidly under humid conditions because of curtailed evaporative cooling (right; evaporative heat loss / metabolic heat production).

Hot Birds
RESEARCH PROJECT
SANBI
Biodiversity for Life
South African National Biodiversity Institute
UNIVERSITEIT VAN PRETORIA
UNIVERSITY OF PRETORIA
YUNIBESITHI YA PRETORIA
